# Asthma patients are at increased risk for adverse events and reoperation following posterior lumbar fusion

**DOI:** 10.1371/journal.pone.0344261

**Published:** 2026-03-26

**Authors:** Julian Smith-Voudouris, Anshu Jonnalagadda, Arya G. Varthi, Jonathan N. Grauer

**Affiliations:** Department of Orthopaedics and Rehabilitation, Yale School of Medicine, New Haven, Connecticut, United States of America; Columbia University Vagelos College of Physicians and Surgeons, UNITED STATES OF AMERICA

## Abstract

**Background:**

Posterior lumbar fusion (PLF) is a common procedure for which patient factors are known to influence outcomes. Although asthma is comorbid for many PLF patients, it has not been assessed for correlation to adverse events following PLF. We used a national administrative database to evaluate the relative odds of adverse events following PLF in patients with versus without asthma.

**Methods:**

Adult patients undergoing primary one- or two-level PLF with or without interbody fusion were identified from 2015 – 2023Q1 PearlDiver datasets. Asthma patients were matched 1:4 based on age, sex, and Elixhauser Comorbidity Index (ECI). The incidence of 90-day adverse events were compared using multivariable logistic regression. Five-year reoperations were compared with Cox proportional hazards modeling. The matched asthma group was then stratified by disease severity for multivariable analysis of 90-day aggregated adverse events.

**Results:**

After matching, 19,078 asthma patients undergoing PLF were compared to 75,838 patients without asthma. Asthma patients were at increased odds of 90-day pulmonary adverse events, (p < 0.0001 for each) as well as non-pulmonary adverse events (p < 0.0001 for each). These associations increased in odds with increasing severity of asthma. Further, asthma patients had significantly higher rates of five-year revision, reflected by lower reoperation free survival compared with non-asthma patients (89.2% versus 92.6%, p < 0.0001).

**Conclusions:**

Over a tenth of patients undergoing PLF were identified as having asthma and were found to be of greater odds of pulmonary and non-pulmonary adverse events (a trend that increased with asthma severity), as well as five-year revisions. Stringent pre-operative optimization and postoperative management strategies should be employed for asthma patients undergoing PLF.

## Introduction

Posterior lumbar fusion (PLF) is commonly considered to address degenerative lumbar pathologies that fail to improve with conservative measures [1,2]. However, this is a moderately invasive procedure following which complications may occur and are of increased incidence for those with defined comorbidities such as diabetes [3], obesity [4], bleeding disorders [5], and need for multi-level fusions [6]. Understanding such relationships is of importance given increased emphasis on improving outcomes and minimizing perioperative complications.

Asthma is a chronic disease that inflames and narrows the airways [7] and is characterized by variable clinical symptoms such as shortness of breath, wheezing, chest tightness, and cough [8]. This is a common condition that affects 300 million people globally and is linked to multiple post-surgical adverse events [9–11]. Existing literature recognizes asthma to be a risk factor for postoperative pulmonary complications such as pneumonia, respiratory failure, and atelectasis following a variety of non-pulmonary surgeries [12,13].

A large database study by Lin *et al.* showed that following a broad range of surgery types, asthma patients had an increased risk of postoperative pneumonia, septicemia, and urinary tract infection post-surgery compared to non-asthmatic patients [10]. Subsequently, within orthopaedic surgery, a recent study by Tveit *et al.* showed that pediatric patients with asthma undergoing surgical repair for tibial shaft fractures had an increased risk of prolonged hospital stay [14]. In adults undergoing total knee arthroplasty, patients with asthma had greater odds of experiencing numerous post-operative complications including pulmonary-related events, as well as infectious-related events such as sepsis and surgical site infection [15]. Related to the spine, a study by Sagi *et al.* demonstrated that asthma is a pertinent risk factor for respiratory compromise and airway obstruction following anterior cervical spine surgery [16]. However, the correlation of asthma with other spine surgeries, such as PLF, has not been reported.

Several mechanisms may increase perioperative risk of asthma patients when undergoing PLF. For example, prone surgical positioning for extended duration can lead to increased intra-abdominal and intrathoracic pressure, contributing to decreased venous return and respiratory compliance [17]. This may predispose patients to atelectasis and ventilation-perfusion mismatch. In patients with asthma, baseline airway inflammation, mucus hypersecretion, and bronchial hyperreactivity [18] amplify these intraoperative stresses. Furthermore, baseline immunologic dysfunction in asthma patients [19] may elevate their risk of infection or delayed recovery outside of the pulmonary domain.

Ultimately, asthma is a common disease, and those with asthma often undergo PLF. The present study aimed to utilize a large, national, administrative database to evaluate the risk of adverse events for asthma patients following PLF relative to matched controls with the goal of defining associated postoperative events and suggest directions for optimizing care algorithms.

## Methods

### Study population

The current study retrieved data from the 2015–2023 quarter 1 (Q1) M170 PearlDiver administrative database (PearlDiver, Colorado Springs, CO). This is a large, national, commercially available, multi-insurance claims database that is well-established for use in spine studies [20–25]. At the time of data collection, Pearldiver included health insurance claims up until 2023 Q1.

As data from this database is in a de-identified and aggregated format, our Institutional Review Board (IRB), Yale IRB, determined that studies utilizing this database are exempt from review. The authors did not have access to information that could identify individual participants during or after data collection. Thus, the authors were not required to receive informed consent prior to accessing the fully anonymized dataset. The data used in this study are owned by the third-party vendor PearlDiver (URL: https://pearldiverinc.com/). The data can be queried using the Bellwether software, which is part of the PearlDiver database. The authors did not receive any special permissions or privileges beyond those granted through payment to the vendor.

Adult patients 18 years or older undergoing single-level posterior lumbar fusion (PLF) with or without interbody fusion, were identified based on Current Procedural Terminology (CPT) codes. A single additional-level fusion code was permitted to limit the study cohort to one- or two-level fusions. Exclusion criteria included those patients less than eighteen years old, patients with 2 + additional level fusion codes, those undergoing concurrent cervical, thoracic, or anterior procedures on the same day, those with a diagnosis of trauma, infection, or neoplasm within the previous 90 days, and those who were not active in the database for 90 days following their procedure.

Of surgical patients who met criteria for the study, patients with and without a history of asthma were then defined based on International Classification of Diseases (ICD)-10 codes (S1 Appendix). Patient age at time of operation, sex, and Elixhauser Comorbidity Index (ECI – a measure of comorbidity burden based on 38 comorbidities) [26] were extracted. Variables for matching were selected a priori based on established associations with postoperative outcomes and overall comorbidity burden. PLF patients with versus without asthma were matched 1:4 using exact matching based on age, sex, and ECI. Age and sex were included as independent predictors of postoperative outcomes, while ECI provides a validated composite measure of medical comorbidity frequently used in database studies to minimize confounding [27–29]. History of smoking within one year prior to surgery and diagnoses of COPD were also abstracted and later controlled for in regression models.

For secondary analyses, the matched asthma cohort was subdivided into groups based on coded asthma severity. Levels of severity match standard descriptions in the current asthma management guidelines [30], and were defined through ICD-10 codes (S1 Appendix). Patients with unspecified asthma severity were retained as a separate comparison group to avoid selection bias associated with restricting analysis to patients with complete coding. Mild intermittent, mild persistent, moderate, severe, and unspecified asthma groups were defined.

### Postoperative outcomes

Ninety-day incidence of adverse events were abstracted using ICD codes with methods previously described [20,21,24,31–33]. Severe adverse events (SAEs) included surgical site infection (SSI), sepsis, deep vein thrombosis (DVT), pulmonary embolism (PE), cardiac event, respiratory failure, re-intubation, and failure to wean off ventilator. Minor adverse events (MAEs) included wound dehiscence, pneumonia, urinary tract infection (UTI), acute kidney injury (AKI), hematoma, transfusion, atelectasis, and pleural effusion. All adverse events (AAEs) were defined as the occurrence of a SAE or MAE.

Ninety-day adverse events were also considered in pulmonary and non-pulmonary categories. Pulmonary adverse events included: pneumonia, atelectasis, respiratory failure, pleural effusion, failure to wean off ventilator, and re-intubation. Non-pulmonary adverse events included: surgical site infections, sepsis, deep vein thrombosis, pulmonary embolism, cardiac event, wound dehiscence, urinary tract infection, acute kidney injury, hematoma, transfusion, ED visits, and hospital readmission. Further identified were markers of 90-day healthcare utilization. These included 90-day rates of emergent department (ED) utilization based on evaluation and management codes and readmissions based on the “Admission” code within PearlDiver.

Five-year survival to reoperations were then identified. These were identified based on the occurrence of any lumbar CPT coding and the re-exploration codes CPT-63042, CPT-63044.

### Statistical analysis

Patient characteristics in the unmatched and matched populations were compared using Student T-Tests, and Chi-squared tests where appropriate. This was done for comparing asthma of varying severity.

Differences in matched asthma versus non-asthma 90-day adverse events were compared by univariable analysis with Chi-squared tests. Comparisons were then done with multivariable logistic regressions for the asthma versus no asthma, as well as varying severity asthma groups. Odds ratios (ORs) and 95% confidence intervals (CIs) were calculated for each adverse event and readmission rates.

Five-year revisions/reoperations were compared using Cox proportional hazards modeling, with hazard ratios (HR) and 95% CIs reported. Model was adjusted for age, sex, ECI, recent smoking history, and COPD. A Kaplan-Meier curve was generated for data visualization.

Statistical analyses were conducted with PearlDiver analytical tools. As a large number of statistical comparisons were performed simultaneously, Bonferroni’s Correction was applied for analyses of 90-day outcomes. Alpha was set at α = 0.0023.

## Results

### Sample cohorts

In total, 181,734 (88.1%) non-asthma patients and 24,589 (11.9%) asthma patients were identified. Those with asthma were on average younger, more likely to be female, and had a greater comorbidity burden than their non-asthma counterparts (Table 1, left columns, p < 0.0001 for each). A greater proportion of asthma patients had a history of smoking during the previous year (24.4% vs. 11.7%) or a prior diagnosis of COPD (29.3% vs. 7.8%) (Table 1, p < 0.0001 for both).

**Table 1:** Patient characteristics of those undergoing posterior lumbar fusion with versus without asthma.

	Unmatched sample			Matched sample (4:1)		
	Non-asthma patients	Asthma patients	P-value	Non-asthma patients	Asthma patients	P-value
**Total**	181,734 (88.1%)	24,589 (11.9%)		75,838 (79.9%)	19,078 (20.1%)	
**Matched Variables**						
**Age** (mean + /- SD)	61.87 + /- 12.38	57.61 + /- 12.23	**P < 0.0001**	59.53 + /- 11.70	59.47 + /- 11.76	P = 0.5277
**Sex**			**P < 0.0001**			P = 0.9386
Female	100,449 (55.3%)	17,657 (71.8%)		51,169 (67.5%)	12,866 (67.4%)	
Male	81,285 (44.7%)	6,932 (28.2%)		24,669 (32.5%)	6,212 (32.6%)	
**ECI** (mean + /- SD)	4.78 + /- 3.40	7.57 + /- 3.79	**P < 0.0001**	6.60 + /- 3.24	6.63 + /- 3.28	P = 0.2232
**Unmatched Variables**						
**Recent Smoking**	21,282 (11.7%)	6,000 (24.4%)	**P < 0.0001**	12,395 (16.3%)	3,947 (20.7%)	**P < 0.0001**
**COPD**	14,252 (7.8%)	7,213 (29.3%)	**P < 0.0001**	8,584 (11.3%)	5,130 (26.9%)	**P < 0.0001**

ECI, Elixhauser Comorbidity Index; COPD, chronic obstructive pulmonary disease.

Matched based on age, sex, and ECI 4:1 non-asthma to asthma patients.

Bold indicates significance of p < 0.05.

After matching 4:1 based on age sex, and ECI, those variables were no longer different. The non-asthma group had 75,838 patients, while the asthma group had 19,078 patients (Table 1, right columns). These matched groups were used for subsequent analyses.

### Postoperative adverse events

Rates of 90-day adverse events following PLF and univariable analysis comparing the matched PLF groups with versus without asthma are shown in Table 2. A striking number of these variables demonstrated increased rates in asthma patients. These included both pulmonary and non-pulmonary related events.

**Table 2:** Univariable analysis of 90-day outcomes in patients undergoing posterior lumbar fusion with versus without asthma.

	Non-asthma patients	Asthma patients	P-value
**Total**	75,838	19,078	
**All adverse events**	11,441 (15.1%)	5,425 (28.4%)	**P < 0.0001**
**Severe adverse events**	5,789 (7.6%)	2,445 (12.8%)	**P < 0.0001**
**Surgical site infection**	1,381 (1.8%)	480 (2.5%)	**P < 0.0001**
**Sepsis**	1,260 (1.7%)	527 (2.8%)	**P < 0.0001**
**Deep vein thrombosis**	1,254 (1.7%)	532 (2.8%)	**P < 0.0001**
**Pulmonary embolism**	745 (1.0%)	297 (1.6%)	**P < 0.0001**
**Cardiac event**	516 (0.7%)	276 (1.4%)	**P < 0.0001**
**Respiratory Failure**	1,329 (1.8%)	842 (4.4%)	**P < 0.0001**
**Re-Intubation**	136 (0.2%)	60 (0.3%)	**P = 0.0003**
Failure to wean off ventilator	95 (0.1%)	35 (0.2%)	P = 0.0668
**Minor adverse events**	8,504 (11.2%)	4,377 (22.9%)	**P < 0.0001**
**Wound dehiscence**	1,640 (2.2%)	543 (2.8%)	**P < 0.0001**
**Pneumonia**	1,198 (1.6%)	1,145 (6.0%)	**P < 0.0001**
**Urinary tract infection**	3,431 (4.5%)	1,979 (10.4%)	**P < 0.0001**
**Acute kidney injury**	2,127 (2.8%)	845 (4.4%)	**P < 0.0001**
**Hematoma**	511 (0.7%)	179 (0.9%)	**P = 0.0001**
**Transfusion**	626 (0.8%)	218 (1.1%)	**P < 0.0001**
**Atelectasis**	1,234 (1.6%)	813 (4.3%)	**P < 0.0001**
**Pleural Effusion**	609 (0.8%)	361 (1.9%)	**P < 0.0001**
Readmission	5,688 (7.5%)	1,460 (7.7%)	P = 0.4848
**ED visits**	11,670 (15.4%)	6,548 (34.3%)	**P < 0.0001**

Bonferroni corrected α = 0.0023.

Bold indicates significance of p < 0.0023.

**Fig 1:**
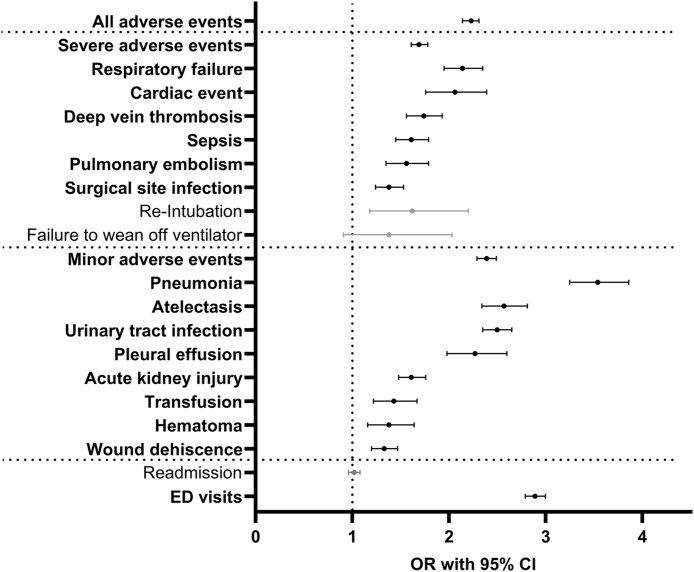
90-day adverse events following posterior lumbar fusion. Forest plot of odds ratios (ORs) and 95% confidence intervals (CIs) of multivariable analysis of 90-day outcomes following PLF patients. Grey denotes nonsignificant odds ratios, black/bold denotes significant odds ratios.

Multivariable analysis controlling for age, sex, and ECI further demonstrated asthma patients undergoing PLF to be at significantly increased odds of experiencing nearly all of the most commonly reported pulmonary and non-pulmonary 90-day adverse events (Table 3, Fig 1). Notably, asthma patients were at increased odds of AAE (OR=2.23), SAE (OR=1.69), and MAE (OR=2.39) (p < 0.0001 for each, Table 3, Fig 1).

**Table 3:** Multivariable analysis of 90-day outcomes controlled for age, sex, ECI, recent smoking history, and COPD in patients undergoing posterior lumbar fusion with versus without asthma.

	Asthma patients [OR (95% CI)]	P-value
**All adverse events**	2.23 (2.14,2.32)	**P < 0.0001**
**Severe adverse events**	1.69 (1.61,1.78)	**P < 0.0001**
**Surgical site infection**	1.38 (1.24,1.53)	**P < 0.0001**
**Sepsis**	1.61 (1.45,1.79)	**P < 0.0001**
**Deep vein thrombosis**	1.74 (1.56,1.93)	**P < 0.0001**
**Pulmonary embolism**	1.56 (1.35,1.79)	**P < 0.0001**
**Cardiac event**	2.06 (1.76,2.39)	**P < 0.0001**
**Respiratory failure**	2.14 (1.95,2.35)	**P < 0.0001**
Re-intubation	1.62 (1.18,2.20)	P = 0.0025
Failure to wean off ventilator	1.38 (0.91,2.03)	P = 0.1151
**Minor adverse events**	2.39 (2.29,2.49)	**P < 0.0001**
**Wound dehiscence**	1.33 (1.20,1.47)	**P < 0.0001**
**Pneumonia**	3.54 (3.25,3.86)	**P < 0.0001**
**Urinary tract infection**	2.50 (2.35,2.65)	**P < 0.0001**
**Acute kidney injury**	1.61 (1.48,1.76)	**P < 0.0001**
**Hematoma**	1.38 (1.16,1.64)	**P = 0.0003**
**Transfusion**	1.43 (1.22,1.67)	**P < 0.0001**
**Atelectasis**	2.57 (2.34,2.81)	**P < 0.0001**
**Pleural effusion**	2.27 (1.98,2.60)	**P < 0.0001**
Readmissions (90 days)	1.02 (0.96,1.08)	P = 0.5165
**ED visits (90 days)**	2.89 (2.79,3.00)	**P < 0.0001**

OR, Odds Ratio; CI, Confidence Interval; COPD, chronic obstructive pulmonary disease.

Bonferroni corrected α = 0.0023.

Bold indicates significance of p < 0.0023.

When considering pulmonary-specific adverse events in order of decreasing odds ratio, the following were identified: pneumonia (OR=3.54), atelectasis (OR=2.57), pleural effusion (OR=2.27), and respiratory failure (OR=2.14) (p < 0.0001 for each, Table 3). Failure to wean (OR=1.38, p = 0.1151) and re-intubation (OR=1.62, p = 0.0025) were not significant following Bonferroni correction.

When considering non-pulmonary related adverse events in order of decreasing odds ratio the following were identified: ED visits (OR=2.89), UTI (OR=2.50), cardiac event (OR=2.06), DVT (OR=1.74), sepsis (OR=1.61), AKI (OR=1.61), PE (OR=1.56), transfusion (OR=1.43), hematoma (OR=1.38, p = 0.0003), SSI (OR=1.38), and wound dehiscence (OR=1.33) (p < 0.0001 for each other than hematoma, Table 3). Only hospital readmissions were not different based on asthma status.

**Fig 2:**
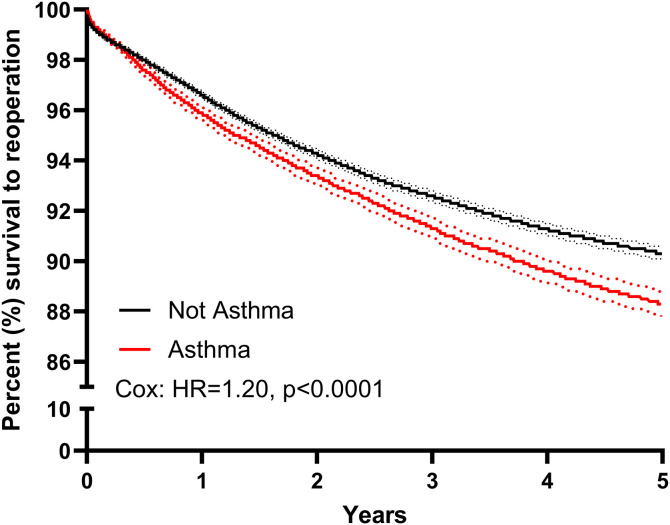
Spinal reoperations in asthma patients. The Kaplan-Meier curve showing five-year instrumentation survival following posterior lumbar fusion comparing the asthma and not asthma matched groups. Adjusted statistics calculated with Cox proportional hazards modeling, controlling for age, sex, ECI, recent smoking history, and COPD. HR, Hazard Ratio.

Asthma patients also had worse five-year survival to reoperation following PLF compared to their non-asthma counterparts. At five years, asthma patients had an 89.2% survival to reoperation versus 92.6% in the non-asthma controls (Hazards ratio [HR]=1.20, 95% CI [1.14–1.27], p < 0.0001, Fig 2).

### Stratification by asthma severity

The matched asthma group was stratified into four levels of disease severity, along with unspecified severity. This resulted in the following: mild intermittent (3,975 patients), mild persistent (1,623 patients), moderate (3,095 patients), severe (871 patients), and unspecified (9,514 patients) (Table 4).

**Table 4:** Patient characteristics of posterior lumbar fusion patients with and without asthma, stratified by severity.

	No Asthma	Mild Intermittent Asthma	Mild Persistent Asthma	Moderate Asthma	Severe Asthma	Unspecified Asthma	P-value
**Total**	75,838	3,975	1,623	3,095	871	9,514	
**Age** (mean + /- SD)	59.53 + /- 11.70	58.57 + /- 12.09	59.33 + /- 11.94	59.28 + /- 11.32	59.56 + /- 11.22	59.93 + /- 11.76	**P < 0.0001**
**Sex**							**P < 0.0001**
Female	51,169 (67.5%)	2,736 (68.8%)	1,119 (68.9%)	2,190 (70.8%)	603 (69.2%)	6,218 (65.4%)	
Male	24,669 (32.5%)	1,239 (31.2%)	504 (31.1%)	905 (29.2%)	268 (30.8%)	3,296 (34.6%)	
**ECI** (mean + /- SD)	6.60 + /- 3.24	6.46 + /- 3.21	6.43 + /- 3.29	6.79 + /- 3.31	7.04 + /- 3.45	6.65 + /- 3.28	**P < 0.0001**
**Recent Smoking**	12,395 (16.3%)	715 (18.0%)	314 (19.3%)	599 (19.4%)	154 (17.7%)	2,165 (22.8%)	**P < 0.0001**
**COPD**	8,584 (11.3%)	767 (19.3%)	397 (24.5%)	955 (30.9%)	367 (42.1%)	2,644 (27.8%)	**P < 0.0001**

ECI, Elixhauser Comorbidity Index; COPD, chronic obstructive pulmonary disease.

Bold indicates significance of p < 0.05.

**Fig 3:**
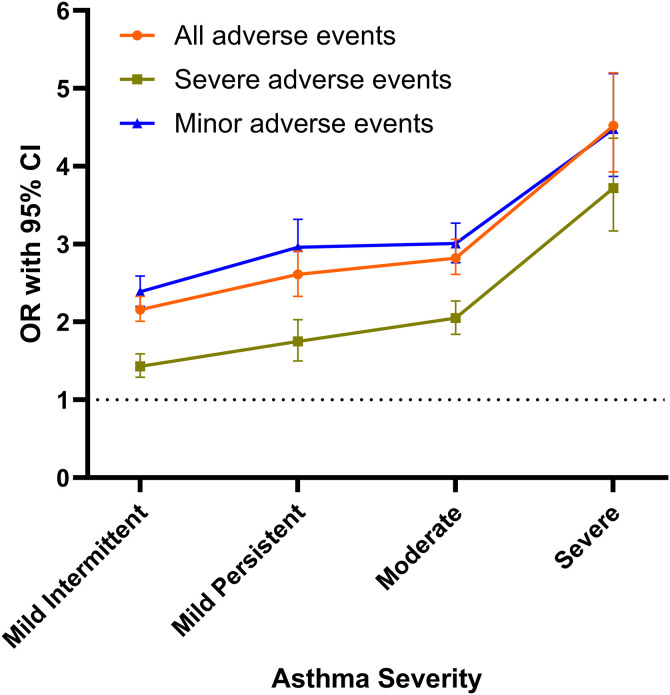
90-day adverse outcomes following posterior lumbar fusion for those with differing levels of asthma severity. Odds ratios (ORs) and 95% confidence intervals (CIs) of multivariable analysis of 90-day outcomes following PLF in patients with mild, moderate, and severe asthma. ORs for adverse events are connected to show change across asthma severity.

Multivariable analysis controlling for age, sex, ECI, smoking, and COPD demonstrated that, compared to patients without asthma, those with any level of asthma severity were at increased odds of all the aggregated adverse events categories following PLF (AAE, MAE, SAE). These odds increased significantly from mild intermittent to severe asthma (Fig 3).

Asthma patients suffered more from AAE (mild intermittent OR=2.16, mild persistent OR=2.61, moderate OR=2.82, severe OR=4.52, unspecified OR=1.88), SAE (mild intermittent OR=1.43, mild persistent OR=1.75, moderate OR=2.05, severe OR=3.72, unspecified OR=1.52), and MAE (mild intermittent OR=2.39, mild persistent OR=2.96, moderate OR=3.01, severe OR=4.48, unspecified OR=1.97) (p < 0.0001 for each, Table 5, Fig 3).

**Table 5:** Multivariable analysis of 90-day outcomes of adult patients with varying severities of asthma relative to those without asthma.

	Mild Intermittent [OR (95% CI)]	P-value	Mild Persistent [OR (95% CI)]	P-value	Moderate Asthma [OR (95% CI)]	P-value	Severe Asthma [OR (95% CI)]	P-value	Unspecified Asthma [OR (95% CI)]	P-value
**All adverse events**	2.16 (2.01,2.33)	P < 0.0001	2.61 (2.33,2.91)	P < 0.0001	2.82 (2.61,3.06)	P < 0.0001	4.52 (3.93,5.20)	P < 0.0001	1.88 (1.78,1.98)	P < 0.0001
**Severe adverse events**	1.43 (1.29,1.59)	P < 0.0001	1.75 (1.50,2.03)	P < 0.0001	2.05 (1.84,2.27)	P < 0.0001	3.72 (3.17,4.36)	P < 0.0001	1.52 (1.42,1.63)	P < 0.0001
**Minor adverse events**	2.39 (2.20,2.59)	P < 0.0001	2.96 (2.63,3.32)	P < 0.0001	3.01 (2.76,3.27)	P < 0.0001	4.48 (3.87,5.19)	P < 0.0001	1.97 (1.86,2.09)	P < 0.0001

OR, Odds Ratio; CI, Confidence Interval.

Reference level = Not asthma.

Bold indicates significance of p < 0.05.

## Discussion

Asthma is a common inflammatory disease that afflicts many patients undergoing PLF. In fact, the current study identified that out of more than 180,000 patients undergoing PLFs, 11.9% had a diagnosis of asthma. Utilizing a large, national, administrative database, the current study assessed perioperative complications and five-year outcomes of those with versus without asthma.

Given its prevalence, it is surprising that asthma has not been investigated in relation to PLF. We hypothesize this absence of investigation may be due to the grouping of asthma in the aging population as chronic obstructive pulmonary disease (COPD). Asthma in older patients is distinct from COPD, with asthma patients exhibiting different patterns of airway inflammation compared to those with COPD, even when both conditions present with airflow obstruction [34]. Furthermore, the prevalence of asthma in populations aged 65 and older is clinically significant, affecting up to 10.9% of adults over 65 years old, who experience symptoms similar to those observed in younger individuals [35–37]. This underscores the necessity of examining asthma as a distinct comorbidity in patients undergoing PLF.

In assessing peri-operative outcomes of those with versus without asthma undergoing PLF, matching was performed to account for potential differential patient characteristics and to delineate independent associations. The current study found asthma patients to be at increased odds of 90-day pulmonary adverse events including pneumonia (OR=3.54), atelectasis (OR=2.57), respiratory failure (OR=2.14), and pleural effusion (OR=2.27). These findings are in parallel to previous literature establishing that asthma patients are prone to perioperative morbidity as a result of their susceptibility to bronchospasm and hypoxemia in response to stimulus, such as endotracheal intubation [11].

Asthma patients undergoing PLF were also found to be increased odds of non-pulmonary adverse events including, ED visits (OR=2.89), UTI (OR=2.50), cardiac event (OR=2.06), DVT (OR=1.74), sepsis (OR=1.61), pulmonary embolism (OR=1.56), AKI (OR=1.61), transfusion (OR=1.43), hematoma (OR=1.38), SSI (OR=1.38), and wound dehiscence (OR=1.33). While existing literature about non-pulmonary postoperative adverse events in asthma patients is limited, other studies have shown similar results. Carlson *et al.* found asthma was a risk factor for experiencing general complication following anterior cervical discectomy and fusion [38], while another database study reported elevated odds of infectious and coagulopathic complications following total knee arthroplasty [15].

Asthma is commonly described as a disease of the airway, yet patients experience systemic inflammation contributing to pathologies of numerous organ systems [39]. The current study identified elevated infectious complications in the asthma population. These may be attributed to asthma patients suffering from chronic impairment of innate and adaptive immunity [40], decreasing their ability to defend against acute infections. In fact, one prior study found patients with asthma versus those without to be at higher risk of infectious events following a broad range of surgeries [10]. With regards to other adverse events, asthma, consequential to chronic inflammatory changes, has been found to promote a prothrombotic state [41], while also being a risk factor for cardiovascular disease and events [42]. Asthma’s association with atherosclerotic cardiovascular disease (ASCVD) is likely due to their shared upregulation of the IL-6 pathway, among other immune related contributors [39,43]. In this context, asthma should be considered a unique source of systemic inflammation associated with experiencing non-pulmonary adverse events following surgery such as PLF.

A pertinent negative is that the current study found no differences in 90-day inpatient hospital readmission in patients with or without asthma. This contrasts with recent literature which has identified asthma as a risk factor for readmission following degenerative lumbar spine surgery [44]. This discrepancy is not fully clear but most likely due to differences in readmission criteria, and the current study’s dependence on inpatient procedural codes to define readmission. More expectedly, the current study found asthma patients more likely to visit the ED post-operatively (OR=2.89).

Asthma patients were also found to be more likely to have a reoperation within five years of surgery, further highlighting their elevated risk following PLF. While the primary indication for reoperation was not clear, this is a relevant finding for patient counseling. In fact, it is noted that the reoperation rates seemed to be diverging over the years following PLF based on the Kaplan-Meier curve.

Upon stratification of the asthma population by disease severity, the current study demonstrated all levels of asthma severity (mild intermittent, mild persistent, moderate, severe, and unspecified) were at higher odds of AAE, SAE, and MAE than those without asthma. Further, those with more severe asthma were at greater odds for experiencing such aggregated adverse events. This progressive increase in adverse outcomes with worsening asthma severity likely reflects the greater physiological burden associated with severe airway disease. It may also be at least partially contributed by more frequent use of immunomodulatory medications in this patient population. However, this relationship remains unclear as several studies have demonstrated continuation or optimization of inhaled corticosteroids can reduce perioperative pulmonary complications in asthma patients [13,45], while more chronic corticosteroid exposure has been linked to impaired wound healing susceptibility to infections [46]. Patients with severe asthma had the largest association with all three categories of aggregated adverse events, with ORs of 4.52, 3.72, and 4.48 respectively. The observed severity-dependent risk gradient highlights the importance of achieving optimal asthma control prior to elective procedures such as PLF. Perioperative risk can be mitigated through ensuring disease stability, continuation of inhaled maintenance therapy, and avoidance of elective surgery during periods after symptom exacerbation [11,13]. For patients with moderate or severe disease, preoperative coordination with pulmonology or anesthesia teams may be beneficial to optimize pulmonary status and perioperative medications [11,18]. Before surgery, patients should be free of wheezing with peak flow greater than 80% of predicted [47]. Attention to intraoperative ventilation strategies and early postoperative pulmonary hygiene may further reduce pulmonary complications in this population [12,17].

Beyond medical management, these findings have implications for risk stratification and patient counseling. Current spine surgery complication risk calculators, such as SpineSage, do not include asthma as distinct risk factor, although other related comorbidities like COPD are included [48]. The current study suggests asthma may represent a clinically significant contributor to perioperative risk following PLF and incorporating asthma status into risk prediction tools could improve estimations of both short- and long-term complications. This may contribute to more nuanced informed consent discussions regarding outcomes following PLF.

The current study does have limitations. As with all studies based on administrative data, results are dependent on the accuracy of the coded data. Specific reasons for reoperation could not be determined, as PearlDiver does not reliably distinguish indications for primary versus revision surgery. Although asthma severity could not be classified for all patients based on ICD-10 coding, these patients were retained as an unspecified asthma group to minimize selection bias and preserve the representativeness of the asthma cohort. Still, heterogeneity within this group may attenuate effect sizes and limits more granular analysis. Additionally, spine specific factors and clinical outcomes could not be assessed. Asthma medication use was also not captured in this analysis and represents a potential source of confounding, particularly when comparing patients with severe disease who are more likely to receive systemic corticosteroids in the perioperative period. Although matching was performed by age, sex, and comorbidity burden, residual confounding may persist from unmeasured variables such as intraoperative ventilation parameters, operative duration, and positioning. While matching by these variables reduces baseline differences, residual confounding from unmeasured clinical factors remains possible and should be considered when interpreting findings. Nor did we compare patients based on anesthesia type as outcomes may be different when utilizing spinal versus general endotracheal intubation anesthesia in patients with lung disease. Future work should incorporate medication-level data and focus on how to implement pre-operative pathways to mitigate adverse events experienced by asthma patients. Still, the statistical power of a large administrative database such as PearlDiver enabled the current study to address a gap in the literature.

In summary, patients with asthma undergoing posterior lumbar fusion were found to have greater odds of numerous pulmonary and non-pulmonary 90-day adverse events (a trend that correlated with asthma severity), as well as worse five-year survival to reoperation. Understanding these elevated risks are imperative for satisfactory pre-operative risk assessment, optimization, and counseling of asthma patients when considering PLF.

## Supporting information

S1 Appendix List of asthma ICD-10 diagnostic codes. (DOCX)
