# Supplementary material for: Asthma patients are at increased risk for adverse events and reoperation following posterior lumbar fusion
Source: PLoS One. 2026 Mar 26;21(3):e0344261. doi: 10.1371/journal.pone.0344261 (PMC13020810; doi:10.1371/journal.pone.0344261)
Supplement: S1 Appendix — (DOCX) [file pone.0344261.s001.docx]

**Appendix – Asthma ICD-10 Codes**

ICD-10-D-J4520: Mild intermittent asthma, uncomplicated

ICD-10-D-J4521: Mild intermittent asthma, acute exacerbation

ICD-10-D-J4522: Mild intermittent asthma, status asthmaticus

ICD-10-D-J4530: Mild persistent asthma, uncomplicated

ICD-10-D-J4531: Mild persistent asthma, acute exacerbation

ICD-10-D-J4532: Mild persistent asthma, status asthmaticus

ICD-10-D-J4540: Moderate persistent asthma, uncomplicated

ICD-10-D-J4541: Moderate persistent asthma, acute exacerbation

ICD-10-D-J4542: Moderate persistent asthma, status asthmaticus

ICD-10-D-J4550: Severe persistent asthma, uncomplicated

ICD-10-D-J4551: Severe persistent asthma, acute exacerbation

ICD-10-D-J4552: Severe persistent asthma, status asthmaticus

**Unspecified Asthma Severity**

ICD-10-D-J45901: Other and unspecified asthma, acute exacerbation

ICD-10-D-J45902: Other and unspecified asthma, status asthmaticus

ICD-10-D-J45909: Other and unspecified asthma, uncomplicated

ICD-10-D-J45991: Cough variant asthma

ICD-10-D-J45998: Other asthma

ICD-10-D-J8283: eosinophilic asthma
